# Vaccine hesitancy among nursing and midwifery undergraduate students in Switzerland: protocol for an online national study

**DOI:** 10.3389/fpubh.2023.1302676

**Published:** 2023-12-12

**Authors:** Audrey Pouvrasseau, Emilien Jeannot

**Affiliations:** ^1^Faculty of Medicine, Institute of Global Health, University of Geneva, Geneva, Switzerland; ^2^Department of Psychiatry, Center for Excessive Gambling, Addiction Medicine (Service), Lausanne University Hospital (CHUV), Lausanne, Switzerland

**Keywords:** vaccine hesitancy, vaccine confidence, HPV, nurse, midwife, student, Switzerland

## Abstract

**Background:**

Vaccine hesitancy is a persistent challenge in public health, exacerbated by the proliferation of anti-vaccine sentiments facilitated by social networks. The COVID-19 pandemic has underscored the importance of addressing vaccine hesitancy, designated by the WHO as a top global health threat. This study explores vaccine hesitancy among nursing and midwifery undergraduate students in Switzerland—a cohort crucial to public health given their future roles as healthcare professionals—with a particular emphasis on the HPV vaccine, which exhibits lower confidence levels compared to other vaccines.

**Methods:**

This study will employ an online questionnaire distributed to nursing and midwifery undergraduate students from various healthcare universities. The questionnaire will collect data on vaccine hesitancy (general confidence in vaccines and specifically in the HPV vaccine), HPV vaccine coverage, socio-demographics, likelihood to recommend vaccines to patients, perception of vaccination education and interest in complementary medicine.

**Conclusion:**

The study’s findings will contribute to our understanding of vaccine hesitancy among nursing and midwifery undergraduate students, providing insights that can inform targeted interventions and education strategies to bolster vaccine confidence among future healthcare professionals, thereby enhancing public health efforts.

## 1 Introduction

Vaccine hesitancy is a phenomenon as old as vaccines themselves, but recent developments in our societies, particularly social networks, provide means for the widespread dissemination of anti-vaccine ideas. The COVID-19 pandemic has also brought this phenomenon to light. While vaccine hesitancy has always existed, it now represents a major challenge and was identified by the World Health Organization (WHO) as one of the top 10 threats to public health in 2019 (1). Experts agree that pandemics like COVID-19 will not be the last humanity will have to face (2). In such a context, ensuring population adherence to public health recommendations and vaccination becomes crucial. It has been established that vaccine hesitancy is a complex, multifactorial phenomenon that varies greatly across regions and time (3–5). As a result, obtaining data tailored to each target population is essential for targeted interventions. Thus, conducting studies on this topic in Switzerland, focusing on specific populations and/or vaccines, is highly relevant.

The concept of “vaccine hesitancy,” often poorly defined in the literature, encompasses a wide range of attitudes, from hesitancy toward vaccination to complete refusal, and this varies depending on the vaccines (3, 6). Its determinants are as varied as the definition is broad. Socio-demographic factors associated with vaccine hesitancy include being female, young (as younger people feels less at risk), having a low level of education, a low level of income, living in a rural area, and belonging to an ethnic minority (3–5, 7). Many other factors come into play: the historical political and socio-cultural context; trust in institutions (policy makers, health system, pharmaceutical industries etc.) and in vaccines (safety, efficacy); the attitude of health professionals toward vaccination; cultural factors, social pressure and religious or personal convictions; the influence of the media, the Internet and social networks; and at a more individual level we find the perceived importance of vaccination and the perceived risk and knowledge about vaccination (3, 6–9). Using vaccination coverage or vaccine uptake as an indicator to measure vaccine hesitancy is not sufficient, as being vaccinated does not exclude the presence of doubts and concerns about vaccination (7).

The lack of a clear definition of “vaccine hesitancy” is also accompanied by a lack of consensus on which tools should be utilized to best measure it, which poses challenges in research and makes it difficult to compare results. To address this issue, the World Health Organization (WHO) established a Strategic Advisory Group of Experts (SAGE) to work on vaccine hesitancy in 2012, with the task to propose a definition of vaccine hesitancy and a model for categorizing its determinants. The WHO-SAGE emphasized the need for the scientific community to use a common definition, and to develop and validate tools for measuring vaccine hesitancy (9, 10). After a thorough mapping of vaccine hesitancy determinants, the WHO-SAGE proposed the following definition:

“Vaccine hesitancy refers to a delay in acceptance or refusal of vaccination despite availability of vaccination services. Vaccine hesitancy is complex and context specific, varying across time, place, and vaccines. It is influenced by factors such as complacency, convenience and confidence” (9).

The adopted definition is rooted in the “3 Cs” model, which identifies complacency, convenience and confidence as the essential components of vaccine hesitancy. In short, convenience is defined as the ease of accessing vaccination services and the practicality of the vaccination process; complacency as the perception of disease risk and the recognition of the importance of immunization; and confidence as:

“trust in (i) the effectiveness and safety of vaccines; (ii) the system that delivers them, including the reliability and competence of the health services and health professionals and (iii) the motivations of policy-makers who decide on the needed vaccines” (9).

Before and since, several survey tools have been developed (11–13), but there is still no agreed-upon measure of vaccine hesitancy. Similarly, the definition developed by the WHO-SAGE is still the subject of debate (14, 15), as illustrated in a recent systematic literature review by Bussink-Voorend et al., with authors proposing to rather define vaccine hesitancy as a state of indecisiveness (16). However, as the WHO-SAGE definition is the most widely accepted to date, and as the tool we chose for this study is based on it, this is the definition we will use here.

Founded in 2010 by Heidi Larson, the Vaccine Confidence Project (VCP) team conducted extensive research to comprehensively examine global confidence issues about vaccination in the general public, the healthcare professionals and pregnant women (3, 10, 17–24). In 2015, Larson et al., highlighted that among the various factors that can modulate vaccine hesitancy as previously defined by the WHO-SAGE, the leading ones were confidence issues (24). More specifically, confidence in safety and efficacy of vaccines, the perceived importance of vaccination (complacency), and religious or personal beliefs were among the key drivers. Based on these studies, the VCP developed the Vaccine Confidence Index™ (VCI) that was tested on a large scale, in 67 countries (22). The VCI has been used to assess vaccine confidence from 2015 to the present day, in over 150 countries worldwide offering a mapping of vaccine confidence around the world and its evolution (25). Since 2018, the VCP has been mandated by the European Union Commission to monitor vaccination confidence within member countries. Switzerland was surveyed in 2018 by the VCP, but not in subsequent years.

The VCI has the advantage of being simple and short, while effectively assessing confidence, making it a very useful tool. It consists of 4 questions that are answered on a 4-point Likert scale, as follows: “overall, I think vaccines are important for children to have; overall, I think vaccines are safe; overall, I think vaccines are effective; vaccines are compatible with my religious, personal or philosophical beliefs.” These questions are then adapted for different vaccines to assess confidence in specific vaccines. A set of questions has also been developed to target healthcare professionals. The utilization of these questions through the Vaccine Confidence Project to map and monitor the fluctuations in confidence across numerous countries worldwide renders it an ideal tool for ensuring the comparability of research results. As Switzerland had been previously surveyed in 2018 in the general population and in 2021 (26) in the healthcare population, we will be able to compare the results of our study with them. For all these reasons, we decided to use the VCI in the present study.

There are limits to the VCI. First, it assesses only a subset of the determinants of vaccine hesitancy. Confidence in vaccination in terms of perceived efficacy, safety, importance, and compatibility with personal beliefs are key determinants of vaccine hesitancy but are not the only ones. Second, although the VCI has been developed on the basis of research studies and tested on a large scale, the tool has not been formally validated. However, a recent study showed an association between the tool measurements and vaccine uptake rates, where a decline in confidence was later translated into a decline in vaccine uptake (27). These results shows that the questions are useful to predict the evolution of vaccine uptake, which is an important information for policy makers.

In this study, we have chosen to target nursing and midwifery students, future healthcare professionals and future key players in vaccination. Research indicates that healthcare professionals play a significant role in influencing their patients’ decisions to get vaccinated (6, 12, 28–30). Vaccine hesitancy also affects these professionals and influences their intention to recommend vaccination to their patients (12, 26, 31–35). A strong association has been observed between healthcare professionals’ confidence in vaccination and the general population’s trust in vaccination (34). Nurses and midwives, in particular, tend to be more hesitant compared to physicians, a difference that could be explained by different training and lack of knowledge regarding vaccination (26, 31, 36). Indeed, studies have shown that there is a difference in the level of knowledge and the presence of more misconceptions among nurses and midwives than among doctors, with the most common barrier being a perceived lack of effectiveness (36, 37).

Therefore, ensuring healthcare professionals’ training and commitment to vaccination plans is essential to combat vaccine hesitancy and maintain adequate population vaccination coverage. Students in particular need to be adequately trained on this subject to be able to promote vaccination later. Most studies on this field of research focus on healthcare workers, but few target nurses and/or midwives in training (38–40). A recent study, with very similar goals to ours, assessed vaccine confidence among healthcare students in South Africa using the VCI (41). In Switzerland, studies targeting the same population evaluated factors influencing HPV vaccination, as vaccination coverage for this vaccine is still too low (42, 43).

Vaccine hesitancy also varies based on the type of vaccine. General confidence in the HPV vaccine tends to be lower than for influenza or Measles, Mumps, and Rubella (MMR) vaccines in the general population, as well as among healthcare professionals (34). Among healthcare professionals, studies have identified gaps in knowledge about the Human Papillomavirus (HPV) vaccine, particularly regarding its functioning and potential benefits (44). A recent study conducted in Italy among university students enrolled in health programs such as medicine, healthcare and pharmacy, showed major gaps in knowledge of HPV infection and preventive measures, and the self-reported vaccination rate was very low (45). This lack of knowledge influences their willingness to get vaccinated, recommend vaccination to their patients, or participate in HPV vaccination recommendation programs (44). These are reasons why we have chosen to focus on the HPV vaccine.

Although there is limited literature on vaccine hesitancy in Switzerland, trends observed align with findings in the global scientific literature. A multicenter study from 2022 examining healthcare professionals’ attitudes toward vaccination showed that Switzerland is one of the countries where nurses and midwives are less confident in the safety, importance or effectiveness of vaccines in general (26). Across the three studied vaccines (COVID-19, HPV, and MMR), the HPV vaccine had the lowest percentage of healthcare professionals inclined to recommend it to their patients (64% in Switzerland). The Federal Office of Public Health (OFSP) has recognized the need to improve healthcare professionals basic education on vaccination (46). A study also revealed healthcare professionals’ interest in further education on the subject due to their relatively low comfort level in advising patients (47). Consequently, surveying nursing and midwifery students will also help assess their perception of the training they receive on vaccination.

Several studies have also demonstrated that the use of complementary or alternative medicine (CAM) by healthcare professionals is often associated with a lower vaccination status, both among practitioners and patients (6, 7). This trend holds true in Switzerland, where practitioners often have a healthcare background (48, 49). Thus, we have also chosen to evaluate this variable in our population.

In conclusion, we have chosen to target a population with a significant role in vaccination and a strong influence on the public. We aim to assess vaccine confidence among these future professionals, who tend to exhibit higher levels of hesitancy according to studies: nurses and midwives. Using the VCI we will assess vaccine confidence in a general sense, vaccine confidence toward the HPV vaccine, and the likelihood to recommend the HPV vaccine to patients as a future healthcare professional. The student status of our population will allow us to assess their perception of the training they receive on vaccination. Additionally, we will evaluate their interest in complementary medicine, determining whether a link exists between vaccine hesitancy and interest in these practices, as illustrated by other studies. We will also ask their vaccination status for the targeted HPV vaccine, to determine whether this population is already vaccinated or if awareness campaigns could be useful to increase vaccination coverage. This data can also be compared with the results of previous studies conducted on this same population to assess any changes in vaccination coverage (42, 43) and with the results of the 2018 VCP survey for Switzerland (25).

## 2 Methods and analysis

### 2.1 Study objectives and design

This study follows a quantitative approach, utilizing an online questionnaire that will target nursing and midwifery undergraduate students from multiple health universities called “High School of Health” (Hautes écoles de Santé, HES) across Switzerland. This research project aims to achieve the following objectives:

- Assess vaccine hesitancy among nursing and midwifery university students in French, German and Italian-speaking Switzerland. This includes assessing their general confidence in vaccines and their confidence specifically in the HPV vaccine.
- Assess HPV vaccine coverage within the same student population.
- Assess likelihood to recommend HPV vaccine to patients as a future healthcare professional.
- Investigate the presence of predictive factors for vaccine hesitancy based on socio-demographic data and interest in complementary medicine.
- Evaluate students’ perceived adequacy of the vaccination education they have received.

### 2.2 Primary and secondary endpoints

For this study, the main variables of interest are vaccine hesitancy and HPV vaccine coverage among nursing and midwifery students in French, German and Italian-speaking Switzerland. To fulfill our objectives, we have developed a questionnaire based on previous research.

To assess vaccine hesitancy and likelihood to recommend HPV vaccine to patients, we selected the Vaccine Confidence Index™ (VCI), focusing on questions relevant to our study’s objectives (50). Additionally, we included two questions assessing HPV vaccination status, adapted from a previous study on the same population (43). This question will allow us to assess both vaccine coverage within the targeted population and whether there is an association between HPV vaccine history and confidence in the HPV vaccine.

Socio-demographic factors such as age, gender, nationality, education level, and interest in complementary medicine may influence vaccine hesitancy and coverage. These factors will be recorded and considered in statistical analyses to identify potential associations with the variables of interest. Such insights will allow comparisons with socio-demographic factors associated with vaccine hesitancy, as documented in relevant studies (4, 5). Identifying these factors (or their absence) could aid targeted awareness campaigns.

To evaluate students’ perceived adequacy of the education they receive on vaccination, we included a question borrowed from a similar US study by Dybsand et al. (38), whose survey questions were based on previously validated templates.

We also added a question to gauge interest in complementary medicine, drawing from studies that explored the link between these practices and vaccine hesitancy (48).

The questionnaire comprises 7 items and a total of 24 questions. It is designed for quick completion (estimated time: 5 min). The complete questionnaire is provided in Supplementary Appendix.

### 2.3 Population and recruitment

The study will involve nursing and midwifery undergraduate university students (HES) in Switzerland. Inclusion criteria are as follows:

- Students enrolled in nursing or midwifery programs at one of the HES institutions of French, German and Italian -speaking Switzerland.
- Participants must be at least 18 years of age.
- Participants should understand the study procedures and willingly participate.

All HES institutions in Switzerland will be contacted for participation. The recruitment process will involve collaboration with program heads at the participating institutions, who will distribute the survey link to students via email. Participation is voluntary. No compensation is planned for participants.

### 2.4 Sample size

The total population of HES midwifery and nursing students in Switzerland is 4,979 (statistics from the Swiss Federal Statistical Office, 2022–2023). The population proportion is based on the results of the 2018 VCP survey, that showed that 52% agreed with the statement “vaccines are safe.” The sample size is calculated to obtain a 95% confidence interval. With a total population of 4,979 students, an alpha of 0.05 and a beta of 0.80, and a 52% vaccine confidence figure, we obtain a sample size of *N* = 357.

### 2.5 Study procedures

The study entails a single questionnaire comprised of 24 questions, self-administered online and taking approximately 5 min to complete. LimeSurvey, a web-based data-collection software, will be used for data collection. The questionnaire link will be sent by program heads, ensuring participant anonymity. Each participant will be assigned a code, with emails and IP addresses stored separately. Data analysis will be performed on coded data, maintaining participant anonymity. A consent form explaining the study’s objectives and procedures will appear at the start of the questionnaire. The duration for data collection will be 20 days, with a reminder email sent after 10 days. The questionnaire will undergo pre-testing with a small sample from the target population before widespread distribution. Participants can withdraw their consent after submitting the questionnaire, provided their data has not been analyzed yet.

### 2.6 Statistical analysis

Data will be analyzed using STATA 17 software, involving descriptive analyses (averages, frequencies, percentages) and multivariate analyses to identify variables significantly associated with vaccine hesitancy. Statistical tests, such as Student’s t-test and chi-squared test, will assess significance at *p* < 0.05.

The VCI questions are answered in a 4-point Likert scale, with the possibility to answer “I do not know.” Responses are recoded to produce just two categories as follows:

- the answers “strongly agree” and “tend to agree” are recoded as “agree”
- the answers “tend to disagree,” “strongly disagree” and “do not know” are recoded as “do not agree.”

The reason for recoding “do not know” as “do not agree” is that respondents who are uncertain or lack the requisite information to formulate definitive responses to these inquiries should be characterized as exhibiting hesitancy.

As the study aims to determine the presence or absence of vaccine hesitancy rather than measure its degree, participants are categorized as either hesitant or non-hesitant, without establishing a specific threshold. For this purpose, responses are recoded into two categories where “agree” reflects confidence in vaccination (or in specific vaccines), and “disagree” reflects a low level of confidence, indicating hesitancy. Results will be presented as the percentage of respondents who “agree” or “disagree” with each item (importance of vaccines, effectiveness of vaccines, safety of vaccines, compatibility of vaccines with one’s beliefs). The same procedure applies to the question set concerning the likelihood of recommending the HPV vaccine. Multivariate analysis will be used to gauge if socio-demographic factors and interest in CAM are associated with a low level of confidence in vaccination, and with an unlikelihood of recommending the HPV vaccine to patients. For the HPV vaccine, the results from the VCI questions will be compared to the vaccine status of the respondents.

As for the set of questions regarding students’ perception of vaccination training received during school, which is also answered on the same Likert scale, we will apply the same method except for the answer “do not know” which will not be recoded as “do not agree.” Indeed, although this question is only asked of final-year students, there is always a chance that teaching on vaccination has not been completed in its entirety depending on the school. The nature of the question is also different from the previous ones. While being unsure about the VCI questions may reflect hesitation and therefore be included in the “disagree” category, we cannot make the same inference about perception of training. Therefore, results for this set of questions will be presented as the percentage of respondents who “agree,” “disagree” or “do not know” with each item.

The results of the study will then be compared with findings from previous studies that surveyed Switzerland using the same questions (25, 26); with other studies surveying the same population in Switzerland using a different questionnaire (42, 43) and with vaccine confidence results from other countries (25, 26, 41).

### 2.7 Handling of missing data

All questions within the online questionnaire are mandatory, thereby ensuring the absence of missing data. However, participants will be given the option to provide responses such as “do not know,” “do not remember,” or “undecided” where such responses are contextually relevant. In the latest version of the VCI, the response “do not know” is coded as “do not agree.” This coding strategy serves the dual purpose of preventing data loss and capturing the nuances of vaccine hesitancy.

## 3 Discussion

Vaccine hesitancy presents a complex and significant challenge to public health efforts worldwide. The impact of misinformation propagated throughout the internet and social media platforms has amplified this concern, undermining vaccination campaigns and threatening herd immunity. In response to this pressing issue, our study will help understanding vaccine hesitancy among nursing and midwifery students in Switzerland, contributing to the broader discourse on addressing vaccination skepticism.

Healthcare professionals play a crucial role in patients’ attitudes toward vaccination. The anticipated results of this study have the potential to drive evidence-based interventions to combat vaccine hesitancy among nursing and midwifery students, before their own beliefs have crystallized. Insights into determinants of hesitancy can help inform improvements in curricula and training programs, ultimately strengthening the role of healthcare professionals as vaccine advocates. Moreover, assessing the HPV vaccine coverage within this population informs the need for awareness campaigns to increase vaccination rates and contribute to public health goals. The inclusion of the HPV vaccine, which often attracts higher levels of hesitancy, adds specificity to our investigation, aligning with the global need to improve HPV vaccine acceptance.

It is essential to acknowledge the limitations of this study protocol. While our design aims to gather valuable insights, cross-sectional studies have inherent limitations in establishing causality. Additionally, self-reported data might introduce response bias when participants provide inaccurate or misleading information in their responses. Voluntary participation can also lead to selection bias, where those most critical of vaccination may be over or under-represented in our sample. Finally, our questionnaire only assesses a subset of the determinants of vaccine hesitancy.

The findings, their implications as well as limitations will be discussed from the perspective of previous studies and future research directions may also be highlighted.

## Ethics statement

The studies involving humans were approved by the Ethics Committee of Cantonal Commission for Research Ethics (CCER) in Geneva, with the registration AO_2023-00037. The studies were conducted in accordance with the local legislation and institutional requirements. The participants provided their written informed consent to participate in this study.

## Author contributions

AP: Conceptualization, Methodology, Writing – original draft, Writing – review & editing. EJ: Conceptualization, Methodology, Supervision, Writing – original draft, Writing – review & editing.
