# Supplementary material for: Vaccine hesitancy among nursing and midwifery undergraduate students in Switzerland: protocol for an online national study
Source: Front Public Health. 2023 Dec 12;11:1302676. doi: 10.3389/fpubh.2023.1302676 (PMC10754524; doi:10.3389/fpubh.2023.1302676)
Supplement: Supplementary file 1 [file Table_1.docx]

1. **Appendix A: survey questionnaire**

| **Items** | **Questions** | **Answers** |
| --- | --- | --- |
| Socio-demographic characteristics | Gender | - Male - Female - Other |
|  | Age | - 18-24 - 25-34 - 35-44 - 45-54 - 55-64 - 65+ |
|  | Nationality | *Choice between a list of countries* |
|  | Have you obtained a diploma in a field other than healthcare, before nursing/midwifery? | - Yes - No |
|  | In which school are you currently studying? | *Choice between a list of schools participating in the study* |
|  | In which health professional education program are you currently enrolled? | - Midwife - Nurse |
|  | What year are you currently in? | - Year 1 - Year 2 - Year 3 |
| Interest in CAM | How interested are you in complementary and alternative medicine such as acupuncture, chiropractic, osteopathy and homeopathy?  *By "interest" we mean as a patient (you have or could use alternative medicine for yourself) and/or as a future healthcare professional (interest in training or including alternative medicine in your future practice).* | - Very interested - Interested - Not interested - Very uninterested - No opinion |
| Vaccinal status | Are you vaccinated against HPV (human papillomavirus)? | - No - Yes - Do not know |
|  | If you answered yes to the previous question, how many doses did you receive? | - 1 dose - 2 doses - 3 doses - Do not remember |
| General vaccine confidence (VCI) | For each statement, indicate the extent to which you agree or disagree:   - Overall, I think vaccines are important for children to have - Overall, I think vaccines are safe - Overall, I think vaccines are effective - Vaccines are compatible with my religious, personal or philosophical beliefs. | - Strongly agree - Tend to agree - Tend to disagree - Strongly disagree - Do not know |
| HPV vaccine confidence (VCI) | For each statement, indicate the extent to which you agree or disagree:   - I think the HPV vaccine is important - I think the HPV vaccine is safe - I think the HPV vaccine is effective - The HPV vaccine is compatible with my religious, personal or philosophical beliefs. |  |
| Healthcare professionals likelihood to recommend vaccines to patients (VCI) | As a health student and later as a professional, how likely are you to recommend HPV vaccination to patients? | - Highly likely - Somewhat likely - Somewhat unlikely - Highly unlikely - Undecided |
| Students perception of vaccination training received during school (Dysband et al.) | My professional program (nursing or midwifery) includes adequate training and/or education in the following topic areas:   - Vaccine preventable diseases - How vaccines work - The safety of vaccines - Vaccine testing and approval process - How to communicate with vaccine-hesitant caregivers/patients | - Strongly agree - Tend to agree - Tend to disagree - Strongly disagree - Do not know |
